# Supplementary figures and images for: Longitudinal Analyses of Mutational Subclonal Architecture and Tumor Subtypes in Recurrent Bladder Cancer
Source: Int J Mol Sci. 2023 May 8;24(9):8418. doi: 10.3390/ijms24098418 (PMC10179737; doi:10.3390/ijms24098418)

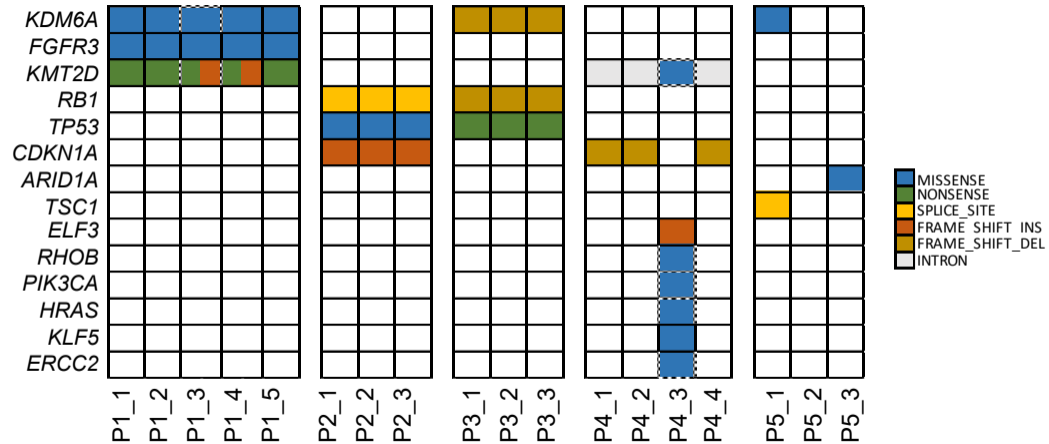

Supplement: Supplementary file 1 [file ijms-24-08418-s001.zip › Supplementary Figure S1.pdf]

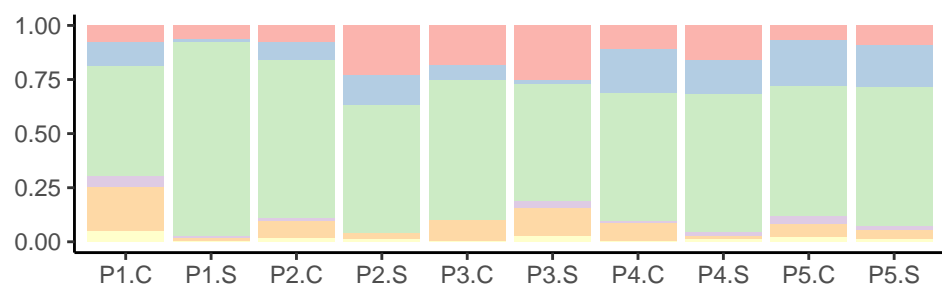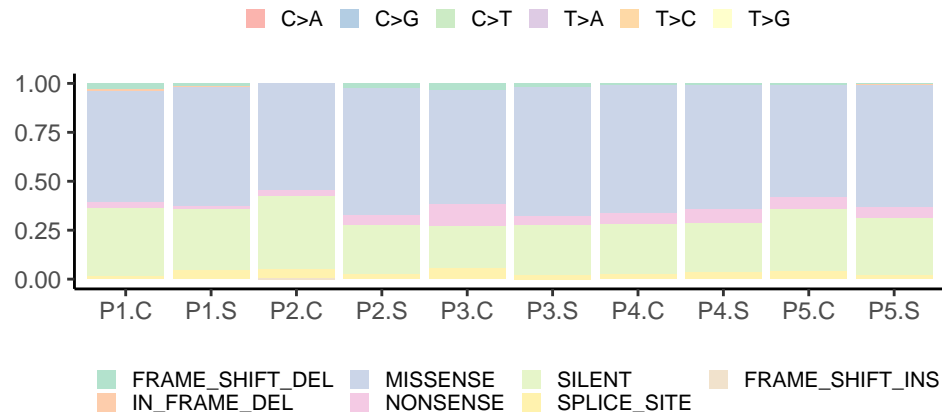

Supplement: Supplementary file 1 [file ijms-24-08418-s001.zip › Supplementary Figure S2.pdf]
